# Supplementary material for: Guided visual search is associated with target boosting and distractor suppression in early visual cortex
Source: Commun Biol. 2025 Jun 11;8:912. doi: 10.1038/s42003-025-08321-3 (PMC12159186; doi:10.1038/s42003-025-08321-3)
Supplement: Supplementary file 1 — Supplementary Material [file 42003_2025_8321_MOESM1_ESM.pdf]

Supplementary Figures

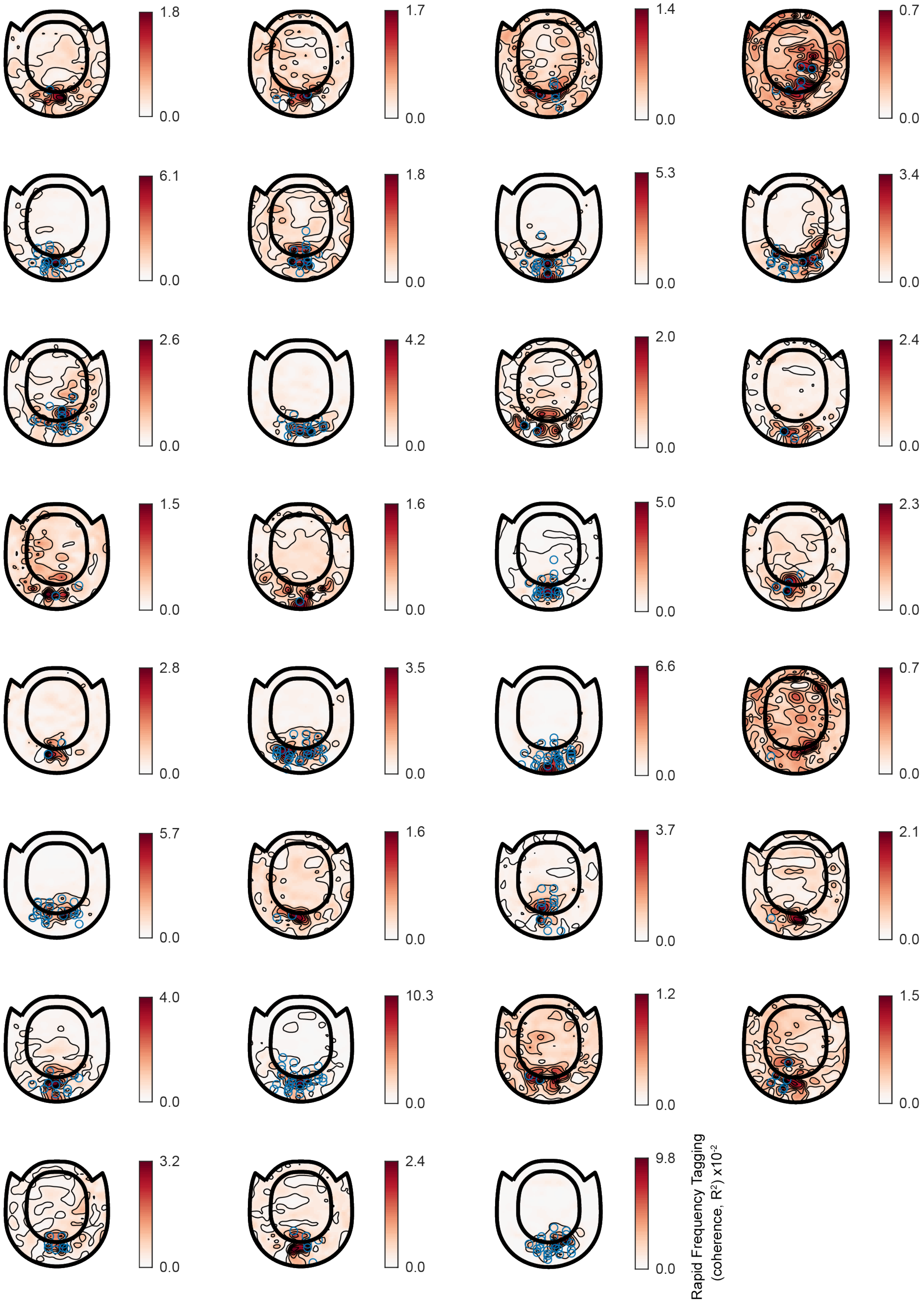

Supplementary Figure 1 Topoplots showing coherence to RIFT at 60 Hz for each participant. RIFT sensors of interest are indicated by the light blue rings.

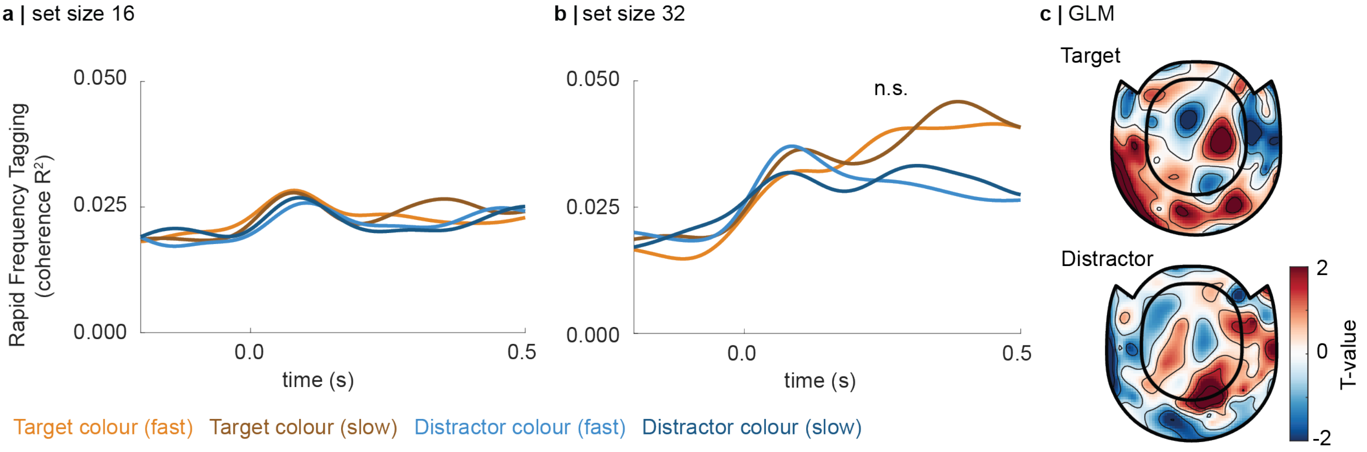

*Supplementary Figure 2 RIFT response as a function of fast and slow trials. Neither a median split on the magnitude-squared coherence (**a**, **b**), nor the GLM approach (**c**) suggest that fast trials are associated with stronger target boosting or distractor suppression. **a** Magnitude-squared coherence associated with the target and distractor for fast and slow trials for set size 16. **b** Same as a for set size 32. **c** T-values associated with the factor reaction time for RIFT response to the target (top) and distractor (bottom) in the unguided search condition, do not indicate stronger target boosting or distractor suppression.*

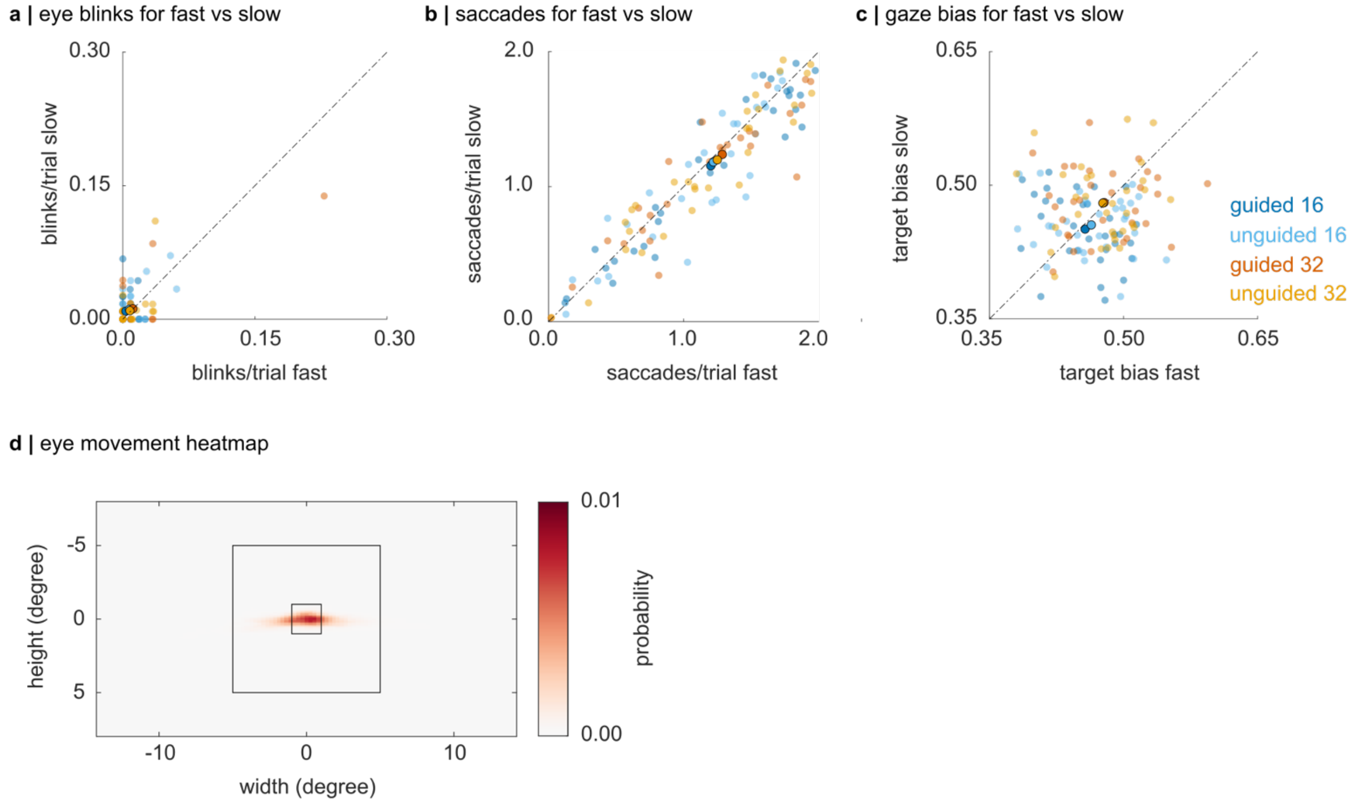

*Supplementary Figure 3 Eye blinks, saccades, and gaze bias towards the target colour for fast vs slow trials. Individual participants are indicated by the opaque scatters, the grandaverage is shown by the solid scatters. **a** Average number of eye blinks in each condition compared for fast and slow trials. A hierarchical regression approach revealed that the factor fast/slow did not add significant explanatory value to the baseline model including subject-specific random effects ($\chi^2(1) = 1.9$, $p = 0.17$). **b** Average number of saccades per trial during the search*

interval in each condition for fast vs slow trials. A hierarchical regression approach indicates a significant main effect for fast vs slow compared to the baseline model ($\chi^2(1) = 3.9$, $p = 0.047$, $\Delta R^2 = 0.0014$), however, the pairwise comparisons did not show any significant differences (see Supplementary analyses) ***c*** *Proportion of the trial the gaze spent near the target colour. A hierarchical regression approach did not show a significant main effect for fast/slow, confirming that participants' gaze tended to spend an equal amount of time within each trial close to stimuli in the target and distractor colour, as indicated by the value of 0.5 ($\chi^2(1) = 0.19$, $p = 0.665$).* ***d*** *Heatmap of eye movements averaged over all participants and trials, in the 0.2 to 0.5s interval after the onset of the search display. The inner box indicates one degree in each direction from the fixation cross (the calibration threshold for the eye tracker), the outer box indicates the search display. The resolution of the heatmap is 0.1 degrees, i.e. 7 pixels. Participants appeared to make mostly horizontal microsaccades, which have been argued to serve to stabilize the retinal image and avoid fading due to neural adaptation (Liang et al., 2005).*

Supplementary Tables

Supplementary Table 1 Hierarchical regression on reaction time, revealing a significant main effect for set size and guided/unguided, but no interaction effect. When fitting the regression models, we assumed that the reaction time data followed a gamma distribution.

**Model**	**AIC**	**X^2**	**p**	**R^2**
$RT = \beta_0 + \varepsilon_{subj}$<br>$\varepsilon_{subj}$ subject-specific random effect	-117.04			0.59
$RT = \beta_0 + \beta_1 X_{set\_size} + \varepsilon_{subj\_setsize}$<br>with $X_{set\_size} = \begin{cases} 1, & set\ size\ 16 \\ 2, & set\ size\ 32 \end{cases}$<br>$\varepsilon_{subj\_setsize}$ subject-specific, set size related random effect	-181.72	70.68	$3x10^{-15}$	0.77
$RT = \beta_0 + \beta_1 X_{set\_size} + \beta_2 X_{guided} + \varepsilon_{subj\_setsize} + \varepsilon_{subj\_guided}$<br>with $X_{guided} = \begin{cases} 0, & unguided \\ 1, & guided \end{cases}$<br>$\varepsilon_{subj\_guided}$ subject-specific, guided/unguided related random effect	-307.57	133.86	$2x10^{-16}$	0.96
$RT = \beta_0 + \beta_1 X_{set\_size} + \beta_2 X_{guided} + \beta_3 X_{interaction} + \varepsilon_{subj\_setsize} + \varepsilon_{subj\_guided}$	-306.08	0.51	0.47	0.96

*Supplementary Table 2 Reaction time contrasts between conditions (dependent sample Wilcoxon signed rank test). SE indicates the standard of the difference between means. Bonferroni corrected, * p < 0.05, ** p < 0.01, *** p < 0.001, **** p < 0.0001*

	**unguided search, set size 16**	**guided search, set size 16**	**unguided search, set size 32**
**guided search, set size 16**	V(30) = 481<br>z = 4.57<br>$p = 8\times10^{-6}$****<br>r = 0.82<br>SE_{diff} = 0.020		
**unguided search, set size 32**	V(30) = 5<br>z = 4.76<br>$p = 6\times10^{-7}$****<br>r = 0.86<br>SE_{diff} = 0.020	V(30) = 0<br>z = 4.86<br>$p = 1\times10^{-11}$****<br>r = 0.87<br>SE_{diff} = 0.03	
**guided search, set size 32**	V(30) = 134<br>z = 2.34<br>p = 0.148 **n.s.**<br>r = 0.4<br>SE_{diff} = 0.019	V(30) = 0<br>z = 4.86<br>$p = 1\times10^{-11}$****<br>r = 0.87<br>SE_{diff} = 0.017	V(30) = 473<br>z= 4.41<br>$p = 4\times10^{-5}$****<br>r = 0.79<br>SE_{diff} = 0.02

Supplementary Table 3 Hierarchical regression on accuracy (d'), revealing a significant main effect for set size and guided/unguided, but no interaction effect.

**Model**	**AIC**	**χ^2**	**p**	**R^2**
$d' = \beta_0 + \varepsilon_{subj}$<br>$\varepsilon_{subj}$ subject-specific random effect	307.10			0.48
$d' = \beta_0 + \beta_{set\_size}X_{set\_size} + \varepsilon_{subj\_setsize}$<br>with $X_{set\_size} = \begin{cases} 1, set\ size\ 16 \\ 2, set\ size\ 32 \end{cases}$	261.56	47.54	$5x10^{-12}$ ****	0.68
$d' = \beta_0 + \beta_1 X_{set_{size}} + \beta_2 X_{guided} + \varepsilon_{subj\_setsize} + \varepsilon_{subj\_guided}$<br>with $X_{guided} = \begin{cases} 0, & unguided \\ 1, & guided \end{cases}$	217.96	45.60	$1x10^{-11}$ ****	0.8
$d' = \beta_0 + \beta_1 X_{set_{size}} + \beta_2 X_{guided} + \beta_3 X_{interaction} + \varepsilon_{subj\_setsize} + \varepsilon_{subj\_guided}$	219.05	0.91	0.34	0.8

Supplementary Table 4 Sensitivity contrasts between conditions (post-hoc dependent sample t-tests, two-sided, Bonferroni-corrected).

	**unguided search, set size 16**	**guided search, set size 16**	**unguided search, set size 32**
**guided search, set size 16**	$t(30) = -5.9$ $p = 8\times10^{-5}$**** $d = -0.84$ $SE_{diff} = 0.11$		
**unguided search, set size 32**	$t(30) = 7$ $p = 6\times10^{-7}$**** $d = 0.8$ $SE_{diff} = 0.096$	$t(30) = 10.6$ $p = 7\times10^{-11}$**** $d = 1.67$ $SE_{diff} = 0.12$	
**guided search, set size 32**	$t(30) = 2.2$ $p = 0.23$ **n.s.** $d = 0.2$ $SE_{diff} = 0.081$	$t(30) = 6.4$ $p = 2\times10^{-6}$**** $d = 0.99$ $SE_{diff} = 0.13$	$t(30) = -5.6$ $p = 3\times10^{-6}$**** $d = -0.55$ $SE_{diff} = 0.09$

Supplementary analyses

Behavioural results: Guided Search is associated with better performance

We predicted that search performance would become worse for more difficult search displays. Indeed, Figure 1c and d, suggests a decrease in reaction time, and an increase in accuracy (d') for *guided* compared to *unguided search* and smaller (16) compared to larger (32) set sizes. We investigated these effects using a hierarchical regression approach with linear mixed models, whereby we consecutively added the factors *set size* and *guided/unguided* (dummy coded as 1 being "guided search") into a model including only subject-related random effects.

To account for the skewed distribution of the reaction time data, we used a gamma distribution to fit the linear mixed models. A model predicting reaction time using the fixed factors *set size* and *guided/unguided*, and the random effects associated with these factors in each participant (AIC = -307.57), was superior to a model predicting reaction time as a function of subject-related random effects and *set size* (AIC = -181.72, $\chi^2(4) = 133.86$, $p < 0.0001$, $R^2 = 0.96$, $\Delta R^2 = 0.22$). This additive model reveals a fixed effect for *set size* ($\beta = 0.180$) and *guided/unguided* ($\beta = - 0.138$). In summary, this demonstrates an increase in reaction time for set size 32 to compared to 16 by about 180ms, and an increase of about 138ms for un*guided* compared to *guided search* (no interaction effect, see Supplementary Table 1).

Analogously, hierarchical regression of accuracy shows that d' is best predicted by an additive model including the factors *set size* ($\beta = - 0.74$) and *guided/unguided* ($\beta = 0.56$, $R^2 = 0.83$) and the subject-specific random effects (see Supplementary Table 2). These results show that accuracy decreases for larger set sizes and increases for *guided* compared to *unguided search*.

The results of all post hoc tests are shown in Supplementary Table 3 and Supplementary Table 4, and indicated in Figure 1c and d. Notably, performance was not significantly different for *unguided search*, set size 16 and *guided search*, *set size 32* (Wilcoxon signed rank test on reaction time : V(30)=134, z = 2.23, p=0.148, r = 0.4, SE = 0.019; sensitivity (d'), dependent sample t-test: t(30)=2.2, p=0.23, d = 0.2, SE = 0.081). This finding is in line with the notion that *guided search* allows the participants to focus their search on items in the target colour, while ignoring the distractor colour.

In summary, the behavioural findings are consistent with a priori expectations, namely an increase in response times with set size, as well as faster responses for *guided* compared to *unguided search*.

RIFT responses for fast compared to slow trials

As the analyses of the RIFT responses in *guided* and *unguided search* revealed a modulation of neuronal excitability in line with the priority map, we asked if successful target boosting and distractor suppression were relevant for performance. We therefore sorted the trials in each condition according to fast and slow responses (median split on reaction time) and compared the respective RIFT signals. Supplementary Figure 2a and b show the RIFT response to the target colour for fast and slow trials (orange and brown line, respectively), and to the distractor colour (light and dark blue for fast and slow trials, respectively) for *guided search*, *set size 16 and 32*. We expected fast trials to be associated with respectively a stronger response to the target colour and a weaker response to the distractor colour, however, we did not find any significant differences between the RIFT responses to targets and distractors for fast vs slow trials. This result was confirmed using the GLM approach on the single-trial coherence, whereby the RIFT response to the target and distractor colours (both set sizes) were submitted to a GLM with the factors constant, time-on-task, and reaction time. Neither the GLM fitted to the target nor the distractor colour suggested a significant correlation between the RIFT response and reaction time (Supplementary Figure 2c).

Ocular artefacts and gaze bias do not relate to RIFT responses

While participants were instructed to perform the task without moving their eyes, we found that some eye movements were present during the search. As enhanced neural processing has been suggested to underlie microsaccades (Liu et al., 2022; Lowet et al., 2018), we investigated if modulated RIFT responses can be explained by differences in ocular artefacts (Supplementary Figure 3).

We divided the trials in the eye tracking data in each condition based on the median reaction time, separately for target present and absent trials in each participant (as described in the main text). Next, we identified the number of blinks and saccades in the first 500 ms after the search display onset (the time interval included in the RIFT analyses) and averaged these over conditions. Note that the threshold of the eye tracker to identify a saccade was set to 0.6°. We again analysed all main and interaction effects using a hierarchical regression approach, by comparing the explanatory value of a model containing the factor fast/slow to the baseline model.

For the average number of blinks during the trial, we find that a model containing the factor *fast/slow* (AIC = -1259.3) did not explain a significantly larger portion of the variance than the baseline model including subject-specific random effects (AIC = -1259.5, $\chi^2(1) = 1.9$, p = 0.17 Supplementary Figure 3a). Using the same approach on the average number of saccades revealed that a model including the predictor *fast/slow* (AIC = - 6.8) could indeed account for a larger portion of the variance than the baseline model (AIC = -4.86, $\chi^2(1) = 3.9$, p = 0.047, ΔR^2 = 0.0014), however, none of the pairwise comparisons reached significance (Supplementary Figure 3b).

To ensure that any eye movements during the search were not over-proportionally directed at the target colour in the fast trials, we binned the eye tracking data into 100ms intervals and identified the stimulus closest to the location of the gaze in each of these bins. The gaze bias towards the target colour was defined as the proportion of time the eyes were directed at a position closest to a stimulus in the target colour. A value of 0.5 indicates that the participant's gaze time on the target and the distractor colour were the same, meaning no bias. Comparing a linear regression model predicting gaze bias as a function of the factor *fast/slow* (AIC = - 8.64.09), with a baseline model (AIC = - 865.9), did not reveal any significant main effects of reaction time on the gaze bias ($\chi^2(1) = 0.19$, p = 0.665, ΔR^2 = 0.0001). This reveals that participants followed the instructions and did not solve the task by moving their eyes towards the target colour (Supplementary Figure 3c).

Supplementary Table 5 Dependent sample t-tests on the number of saccades for fast vs slow trials reveals no significant effects of reaction time on saccades. The p-values are Benjamini-Hochberg corrected, however, none of the uncorrected p-values reached significance either.

**unguided search, *set size 16***	**guided search, *set size 16***	**unguided search, *set size 32***	**guided search, *set size 32***
t(30) = - 0.92<br>p = 0.365	t(30) = - 1.7<br>p = 0.304	*t(30)* = -1.47<br>p = 0.304	*t(30)* = -1.21<br>p = 0.313
