# Supplementary material for: Guided visual search is associated with target boosting and distractor suppression in early visual cortex
Source: Commun Biol. 2025 Jun 11;8:912. doi: 10.1038/s42003-025-08321-3 (PMC12159186; doi:10.1038/s42003-025-08321-3)
Supplement: Supplementary file 2 — Description of Additional Supplementary Files [file 42003_2025_8321_MOESM2_ESM.docx]

Description of Additional Supplementary Files

**File name:** Supplementary Data

**Description:** The source data to create all figures.
