# Supplementary material for: Guided visual search is associated with target boosting and distractor suppression in early visual cortex
Source: Commun Biol. 2025 Jun 11;8:912. doi: 10.1038/s42003-025-08321-3 (PMC12159186; doi:10.1038/s42003-025-08321-3)
Supplement: Supplementary file 3 — Supplementary Data [file 42003_2025_8321_MOESM3_ESM.zip › supplementary_data_fig1-3/README.rtf]

Source datafig1_performance_condi.xlsReaction time (RT) and sensitivity (d prime) per condition.See sheet ‘key’ for explanation of rows.Shown in Fig 1 c and d.fig2a_suppfig1_rift_60Hz_subj.matSingle-subject coherence values for 60 Hz RIFT over all conditions.Used for single subject topoplots (Supp. Fig 1) and grand average (Fig. 2a)Requires conversion to matlab struct in fieldtrip format. See https://github.com/katduecker/visual_search_rift/blob/main/matlab_scripts/coherence/a2_plot_SNR.mfig2b_rift_spectra.xlsSingle subject spectra of RIFT response (coherence). Grandaverage shown in Fig 2b.Columns: subject id, and frequenciesfig2bc_rift_spectrum_tfr.matSingle subject coherence spectra for RIFT at 60 and 67 Hz. TFR averaged over all conditions.fig2d_coherence_set16.xlsxfig2e_coherence_set32.xlsxSingle subject time series of coherence used to calculate average shown in Fig 2d and e.fig3_tboost_dsuppr_cluster_coh_set16.matfig3_tboost_dsuppr_cluster_coh_set32.matResults of cluster-based dependent sample t-tests shown in Fig. 3b and c. Requires field trip. See https://github.com/katduecker/visual_search_rift/blob/main/matlab_scripts/glm_spec/b2_RIFT_RT_stats.msuppfig2_coherence_fast_slow.xlsxSingle subject time series of coherence used to calculate grand average presented in Supp Fig 2 a b.suppfig2_tboost_dsuppr_cluster_coh_set16.matsuppfig2_tboost_dsuppr_cluster_coh_set32.matResults of cluster-based permutation t-test on GLM regressors shown in Supp Fig. 2c. Requires fieldtrip. See https://github.com/katduecker/visual_search_rift/blob/main/matlab_scripts/glm_spec/b2_RIFT_RT_stats.msuppfig3_gaze_fast_slow.xlsxEye blinks, number of saccades, and gaze bias as a function of fast vs slow trials shown in Supp Fig. 3a-c.suppfig3_subj_heatmap.mat3d array (subj x x-coordinate x y-coordinate) of indvidual heat maps, used to calculate grand average shown in Supp Fig. 3d
