# Supplementary material for: Guided visual search is associated with target boosting and distractor suppression in early visual cortex
Source: Commun Biol. 2025 Jun 11;8:912. doi: 10.1038/s42003-025-08321-3 (PMC12159186; doi:10.1038/s42003-025-08321-3)
Supplement: Supplementary file 5 — Reporting Summary [file 42003_2025_8321_MOESM5_ESM.pdf]

nature portfolio

Corresponding author(s): COMMSBIO-25-4392-T

Last updated by author(s): May 29, 2025

Reporting Summary

Nature Portfolio wishes to improve the reproducibility of the work that we publish. This form provides structure for consistency and transparency in reporting. For further information on Nature Portfolio policies, see our Editorial Policies and the Editorial Policy Checklist.

Statistics

For all statistical analyses, confirm that the following items are present in the figure legend, table legend, main text, or Methods section.

n/a	Confirmed	
☐	☒	The exact sample size (n) for each experimental group/condition, given as a discrete number and unit of measurement
☐	☒	A statement on whether measurements were taken from distinct samples or whether the same sample was measured repeatedly
☐	☒	The statistical test(s) used AND whether they are one- or two-sided<br>*Only common tests should be described solely by name; describe more complex techniques in the Methods section.*
☐	☒	A description of all covariates tested
☐	☒	A description of any assumptions or corrections, such as tests of normality and adjustment for multiple comparisons
☐	☒	A full description of the statistical parameters including central tendency (e.g. means) or other basic estimates (e.g. regression coefficient) AND variation (e.g. standard deviation) or associated estimates of uncertainty (e.g. confidence intervals)
☐	☒	For null hypothesis testing, the test statistic (e.g. F, t, r) with confidence intervals, effect sizes, degrees of freedom and P value noted<br>*Give P values as exact values whenever suitable.*
☐	☒	For Bayesian analysis, information on the choice of priors and Markov chain Monte Carlo settings
☐	☒	For hierarchical and complex designs, identification of the appropriate level for tests and full reporting of outcomes
☐	☒	Estimates of effect sizes (e.g. Cohen's d, Pearson's r), indicating how they were calculated

Our web collection on statistics for biologists contains articles on many of the points above.

Software and code

Policy information about availability of computer code

Data collection	The softwares, website links and other data collection tools were stated in the text (result or methods sections)
Data analysis	The softwares, website links and other data analysis tools were stated in the text (result or methods sections)

For manuscripts utilizing custom algorithms or software that are central to the research but not yet described in published literature, software must be made available to editors and reviewers. We strongly encourage code deposition in a community repository (e.g. GitHub). See the Nature Portfolio guidelines for submitting code & software for further information.

Data

Policy information about availability of data

All manuscripts must include a data availability statement. This statement should provide the following information, where applicable:

- Accession codes, unique identifiers, or web links for publicly available datasets
- A description of any restrictions on data availability
- For clinical datasets or third party data, please ensure that the statement adheres to our policy

Provide your data availability statement here.

Research involving human participants, their data, or biological material

Policy information about studies with human participants or human data. See also policy information about sex, gender (identity/presentation), and sexual orientation and race, ethnicity and racism.

Reporting on sex and gender	Unless elsewhere indicated, the genders of animals used in this study were 50% male and 50% female
Reporting on race, ethnicity, or other socially relevant groupings	Race, ethnicity or other social relevant concerns were compliant with policies
Population characteristics	Population characteristics were compliant with policies
Recruitment	No self-selection bias for participant recruitment
Ethics oversight	The animal experiment protocols were approved by IACUC of Second Affiliated Hospital of Army Medical University

Note that full information on the approval of the study protocol must also be provided in the manuscript.

Field-specific reporting

Please select the one below that is the best fit for your research. If you are not sure, read the appropriate sections before making your selection.

☒ Life sciences ☐ Behavioural & social sciences ☐ Ecological, evolutionary & environmental sciences

For a reference copy of the document with all sections, see nature.com/documents/nr-reporting-summary-flat.pdf

Life sciences study design

All studies must disclose on these points even when the disclosure is negative.

Sample size	The sample size was determined by other relevant publications or requirements from FDA or NMPA for IND application
Data exclusions	No data exclusion from analysis
Replication	All attempts at replication were successful
Randomization	Samples were allocated randomly unless elsewhere indicated in the manuscript (results or methods sections)
Blinding	Proper single- or double-blinded allocations were arranged during the study

Reporting for specific materials, systems and methods

We require information from authors about some types of materials, experimental systems and methods used in many studies. Here, indicate whether each material, system or method listed is relevant to your study. If you are not sure if a list item applies to your research, read the appropriate section before selecting a response.

Materials & experimental systems

n/a	Involved in the study	
☐	☒	Antibodies
☐	☒	Eukaryotic cell lines
☒	☐	Palaeontology and archaeology
☐	☒	Animals and other organisms
☒	☐	Clinical data
☒	☐	Dual use research of concern
☒	☐	Plants

Methods

n/a	Involved in the study	
☒	☐	ChIP-seq
☐	☒	Flow cytometry
☒	☐	MRI-based neuroimaging

Antibodies

Antibodies used	E1A (sc-58658, Santa Cruz), E1B (PVV02401, AntibodySystem), H6PD (sc-377180, Santa Cruz), caspase-3 (sc-7272, Santa Cruz), Lamin A/C (4777S, Cell Signaling Technology), TdTomato (20163S, Cell Signaling Technology) and GAPDH (sc-47724, Santa Cruz)
Validation	E1A website: https://www.scbt.com/p/adenovirus-5-e1a-antibody-m58

Validation

E1B website: https://www.antibodysystem.com/product/2318.html
H6PD website: https://www.scbt.com/p/h6pd-antibody-c-10
caspase-3 website: https://www.scbt.com/p/caspase-3-antibody-e-8
Lamin A website: https://www.cellsignal.com/products/primary-antibodies/lamin-a-c-4c11-mouse-mab/4777
TdTomato website: https://www.cellsignal.com/products/primary-antibodies/tdtomato-e3g5l-rabbit-mab/20163
GAPDH website: https://www.scbt.com/p/gapdh-antibody-0411

Eukaryotic cell lines

Policy information about cell lines and Sex and Gender in Research

Cell line source(s)	HEK293 (ATCC CRL-1573), HeLa (ATCC CCL-2), A549 (ATCC CCL-185)
Authentication	Authentication process are on ATCC websites of the cells: HEK293 (https://www.atcc.org/products/crl-1573); HeLa (https://www.atcc.org/products/ccl-2); A549 (https://www.atcc.org/products/ccl-185)
Mycoplasma contamination	Informations about any contaminations are on ATCC websites of the cells as listed above
Commonly misidentified lines (See ICLAC register)	No

Animals and other research organisms

Policy information about studies involving animals; ARRIVE guidelines recommended for reporting animal research, and Sex and Gender in Research

Laboratory animals	C57BL/6 mice (000664, Jackson Laboratory), 8-10 weeks of age, 50% male and 50% female
Wild animals	No wild animal used in the study
Reporting on sex	Genders were evenly distributed: 50% male and 50% female
Field-collected samples	No field-collected samples in this sutdy
Ethics oversight	Animal protocols were approved by IACUC of the second Affiliated Hospital of Army Medical University

Note that full information on the approval of the study protocol must also be provided in the manuscript.

Plants

Seed stocks	No plants in this study
Novel plant genotypes	No plants in this study
Authentication	No plants in this study

Flow Cytometry

Plots

Confirm that:

☒ The axis labels state the marker and fluorochrome used (e.g. CD4-FITC).

☒ The axis scales are clearly visible. Include numbers along axes only for bottom left plot of group (a 'group' is an analysis of identical markers).

☒ All plots are contour plots with outliers or pseudocolor plots.

☒ A numerical value for number of cells or percentage (with statistics) is provided.

Methodology

Sample preparation	The HEK293 cells were collected by centrifugation at 800 rpm, 5min and resuspended in PBS+10% FBS for sorting

Instrument	BD FACSAria™ III cell sorter
Software	BD FACSDive software for data analysis
Cell population abundance	First, the live cell population was identified by FSC > 150 and SSC >150. Next, to further specifically sort the GFP positive cells, the subgroup of cells with FITC channel signal higher than 10^3 were sorted. Please refer to manuscript Figure 1E for details.
Gating strategy	First, the live cell population was identified by FSC > 150 and SSC >150. Next, to further specifically sort the GFP positive cells, the subgroup of cells with FITC channel signal higher than 10^3 were sorted. Please refer to manuscript Figure 1E for details.

☒ Tick this box to confirm that a figure exemplifying the gating strategy is provided in the Supplementary Information.
